# The Major Immediate-Early Protein IE2 of Human *Cytomegalovirus* Is Sufficient to Induce Proteasomal Degradation of CD83 on Mature Dendritic Cells

**DOI:** 10.3389/fmicb.2017.00119

**Published:** 2017-02-01

**Authors:** Christiane S. Heilingloh, Linda Grosche, Mirko Kummer, Petra Mühl-Zürbes, Lisa Kamm, Myriam Scherer, Melanie Latzko, Thomas Stamminger, Alexander Steinkasserer

**Affiliations:** ^1^Department of Immune Modulation, University Hospital ErlangenErlangen, Germany; ^2^Institute for Clinical and Molecular Virology, University of Erlangen-NurembergErlangen, Germany

**Keywords:** dendritic cells, HCMV, CD83, proteasomal degradation, IE2

## Abstract

Human cytomegalovirus (HCMV) is the prototypic beta-herpesvirus and widespread throughout the human population. While infection is asymptomatic in healthy individuals, it can lead to high morbidity and mortality in immunocompromised persons. Importantly, HCMV evolved multiple strategies to interfere with immune cell function in order to establish latency in infected individuals. As mature DCs (mDCs) are antigen-presenting cells able to activate naïve T cells they play a crucial role during induction of effective antiviral immune responses. Interestingly, earlier studies demonstrated that the functionally important mDC surface molecule CD83 is down-regulated upon HCMV infection resulting in a reduced T cell stimulatory capacity of the infected cells. However, the viral effector protein and the precise mechanism of HCMV-mediated CD83 reduction remain to be discovered. Using flow cytometric analyses, we observed significant down-modulation of CD83 surface expression becoming significant already 12 h after HCMV infection. Moreover, Western bot analyses revealed that, in sharp contrast to previous studies, loss of CD83 is not restricted to the membrane-bound molecule, but also occurs intracellularly. Furthermore, inhibition of the proteasome almost completely restored CD83 surface expression during HCMV infection. Results of infection kinetics and cycloheximide-actinomycin D-chase experiments, strongly suggested that an HCMV immediate early gene product is responsible for the induction of CD83 down-modulation. Consequently, we were able to identify the major immediate early protein IE2 as the viral effector protein that induces proteasomal CD83 degradation.

## Introduction

Dendritic cells (DCs) are known as professional antigen presenting cells because of their ability to prime naïve T cells and thereby to induce effective antiviral immune responses. As immature DCs they lay in wait in almost all peripheral tissues until antigen is encountered and taken up. As a consequence DCs begin to mature and migrate along a chemokine gradient toward the draining lymph nodes for T cell activation. During maturation, expression of MHC class I and II as well as of co-stimulatory molecules, such as CD80, CD86 is upregulated (Banchereau and Steinman, 1998). Furthermore, expression of the surface molecule CD83, which is one of the best maturation marker for DCs, is strongly induced (Zhou and Tedder, 1996). In addition, CD83 is also expressed on subsets of activated T cells, B cells, as well as on neutrophils, thymus epithelial cells and on regulatory T cells (Kozlow et al., 1993; Fujimoto et al., 2002; Iking-Konert et al., 2002; Wolenski et al., 2003; Kreiser et al., 2015). Besides the above mentioned membrane bound CD83 molecule (mCD83) also a soluble form of CD83 (sCD83) has been described. This sCD83 is released from activated DCs as well as from B cells and is detectable at low levels in sera of healthy individuals (Hock et al., 2001) and at elevated levels in patients suffering from hematological malignancies (Hock et al., 2004). The immunosuppressive effect of sCD83 was shown in different animal models for autoimmune diseases as well as in transplantation models (Zinser et al., 2004; Ge et al., 2010; Lan et al., 2010; Bock et al., 2013; Eckhardt et al., 2014).

In contrast to sCD83, mCD83 expressed on mDCs has been suggested to have co-stimulatory properties, since knockdown experiments of mCD83 using an siRNA approach in DCs resulted in a significantly reduced T cell stimulatory capacity of these DCs (Aerts-Toegaert et al., 2007; Prechtel et al., 2007). Therefore, with respect to these studies showing the functional importance of CD83, it is not surprising that several viruses, especially herpesviruses, target CD83 upon infection of mDCs. Herpes simplex virus type 1 (HSV-1), varicella-zoster virus and also the human cytomegalovirus (HCMV) reduce the surface expression of CD83 significantly. This in turn results in an inhibited T cell proliferative capacity and therefore in a reduced antiviral immune response (Kruse et al., 2000; Morrow et al., 2003; Senechal et al., 2004).

Human cytomegalovirus is the most complex representative within the herpesviruses and belongs to the *Betaherpesvirinae* subfamily. Characteristic features of HCMV are the high species specificity, a prolonged replication cycle, formation of enlarged cells (cytomegaly) and the establishment of latent infections in CD14^+^ monocytes and their CD34^+^ myeloid progenitor cells (Whitley, 1996; Jackson et al., 2011; Rossetto et al., 2013). In healthy immunocompetent adults primary infection is usually asymptomatic. However, primary HCMV infection or reactivation from latency in immunocompromised individuals can lead to the development of life- and sight-threatening diseases including hepatitis, pneumonitis, retinitis, encephalitis, or gastroenteritis (Rubin, 1998; Landolfo et al., 2003). Moreover, HCMV is presently the leading cause of virus-associated birth defects including mental retardation, blindness, or hearing loss (Chavanas, 2016). HCMV infects a remarkably broad cell range including parenchymal cells and connective tissue cells of almost any organ as well as various hematopoietic cell types. Targets for viral replication are predominantly fibroblasts and smooth muscle cells as well as epithelial and endothelial cells (Sinzger, 2008; Revello and Gerna, 2010). Viral gene expression occurs in a temporally regulated cascade that is subdivided into three sequential phases termed immediate early (IE), early (E), and late (L; Wathen and Stinski, 1982).

Specifically, the tegument protein pp71, which is released into the host cell during viral entry, is capable to induce expression of IE genes, particularly UL122 (IE2) and UL123 (IE1), which are encoded by the major IE locus (Kalejta, 2008). Both, IE1 and IE2, cooperate in initiation of viral early and late gene expression and consequently play an important role in regulation of HCMV infection (Malone et al., 1990). While IE2 is absolutely essential for progression of the infectious cycle from IE to E phase, HCMV mutants defective in IE1 expression show a severe growth defect (Greaves and Mocarski, 1998). Therefore it is thought that IE2 plays a crucial role in triggering the lytic replication cycle of HCMV (Marchini et al., 2001). During reactivation the infection is under control of the immune system but the virus cannot be eliminated completely. However, besides the establishment of latency HCMV has developed additional strategies to escape the host’s immune response. Examples are the degradation of newly synthesized MHC class I heavy chains and the down-modulation of MHC class II molecules (Jones et al., 1995; Hegde et al., 2003). Down-regulation of MHC class I and II molecules restricts presentation of viral antigens and thus limits elimination of the virus by the human immune system (Noriega et al., 2012). An additional and different escape mechanism of HCMV has been reported by Senechal et al. (2004). This group postulated that HCMV infection of mDCs mediates shedding of the immunosuppressive soluble CD83 molecule into the supernatant which subsequently leads to a reduction of DC-mediated antiviral T cell stimulation (Senechal et al., 2004).

In contrast, in the present study we show that HCMV infection of mDCs leads to the degradation of CD83 in a proteasome dependent manner. Furthermore, we were able to show that the immediate early protein 2 (IE2) is sufficient to induce CD83 degradation even in the absence of any other viral factor.

## Materials and Methods

### Virus Strains, Virus Preparation, and Virus Titration

In these studies the HCMV TB40E/IE2-EYFP strain expressing IE2-EYFP fusion protein (Wagenknecht et al., 2015) and the HCMV TB40E/UL84Pr-luc expressing luciferase under the control of the UL84 promoter were used.

Virus stocks were prepared by infection of human foreskin fibroblasts (HFFs) with the respective viral strain. HFFs were cultured in Dulbecco’s minimal essential medium (DMEM; Lonza, Basel, Switzerland) supplemented with 10% FCS (PAA, Cölbe, Germany), 2 mM L-glutamine (Lonza), 100 U/ml penicillin (Lonza), and 100 U/ml streptomycin (Lonza) until a complete cytopathic effect was achieved. Cellular debris was removed by centrifugation and virus containing cell culture supernatant was stored -80°C.

Virus titers for HCMV strains were determined as described elsewhere (Andreoni et al., 1989; Wagenknecht et al., 2015).

### Generation of Mature Dendritic Cells (mDCs)

Monocyte-derived DCs were generated as described previously (Heilingloh et al., 2014). In brief, peripheral blood mononuclear cells (PBMCs) were isolated from different healthy donors using a Lymphoprep gradient (Nycomed Pharma AS, Oslo, Norway) and afterward monocytes were separated using plastic adherence. By addition of 800 U/ml granulocyte macrophage-colony stimulating factor (GM-CSF; CellGenix, Freiburg, Germany) and 250 U/ml IL-4 (Milteny, Bergisch-Gladbach, Germany) monocytes differentiated to immature DCs. Maturation was induced by adding 10 ng/ml TNF-α (Beromun, Boehringer Ingelheim, Germany), 1 mg/ml prostin E2 (PGE2; Pfizer, NY, USA); 200 U/ml IL-1β (CellGenix), 40 U/ml GM-CSF, 1000 U/ml IL-6 (CellGenix), and 250 U/ml IL-4 to the medium. The maturation status was controlled by flow cytometry, and mDCs were used for further experiments.

### Infection Procedure

For infection of mDC, 5 × 10^5^ cells, as a maximum, were washed with PBS and resuspended directly in virus stock at a multiplicity of infection (MOI) of 2. For mock-infected samples, mDCs were resuspended in the equal volume of DMEM supplemented with 10% FCS, 2 mM L-glutamine, 100 U/ml penicillin, and 100 mg/ml streptomycin. Infection was performed in a microcentrifuge at 23 × *g* for 1 h at room temperature. Subsequently, cells were transferred into a 24 well plate and Roswell Park Memorial Institute medium (RPMI 1640, Lonza) supplemented with 1% autologous serum (Sigma Aldrich, Deisenhofen, Germany), 10 mM HEPES (Lonza), 2 mM L-glutamine, 100 U/ml penicillin, 100 mg/ml streptomycin, 40 U/ml GM-CSF, and 250 U/ml IL-4 was added to a final volume of 0.5 ml.

### Immunofluorescence Confocal Microscopy

Mock- or HCMV-infected mDCs were allowed to adhere on poly-L-lysin (Sigma Aldrich) coated glass cover slips, fixed with 4% paraformaldehyde and permeabilized using 0.2% Triton-X-100. Blocking was performed using 1% BSA in PBS. Antibodies used for Immunofluorescence staining: anit-CD83 (HB15a; Beckmann Coulter, Brea, USA); AlexaFluor647-conjugated secondary antibody (Invitrogen, Carlsbad, CA, USA). For mounting and additional nuclear staining Roti^®^-Mount FluorCare DAPI (Carl Roth) was used. Confocal microscopy was performed using a LSM780 microscope (Zeiss, Oberkochen, Germany).

### Inhibitors

Proteasome inhibitors MG-132 (Enzo Life Sciences, Lörrach, Germany), Epoxomicin (Enzo Life Sciences), and Bortezomib (Santa Cruz Biotech. Inc, Heidelberg, Germany) were added 1 h post-infection (hpi) at a final concentration of 10, 5, and 2 μM, respectively.

Cycloheximide (CHX) and actinomycin D (ActD; Sigma Aldrich) were used at a final concentration of 100 and 5 μg/ml, respectively.

### Flow Cytometric Analysis and Cell Sorting

Detection of YFP expression of HCMV and changes in expression of cell surface molecules were analyzed by flow cytometry using antibodies specific for CD83 (clone HB15e; BD Biosciences, Heidelberg, Germany), CD80 (clone L307.4; BD Biosciences), and HLA-DR (clone G46-6, BD Biosciences). To assure analysis of living cells, samples were additionally stained using LIVE/DEAD^®^ Fixable Violet Dead Cell Stain Kit (Life Technologies, Carlsbad, CA, USA). Unstained cells were used as negative control.

Cells were sorted based on their YFP expression using a BD Aria FACS Sorter (BD Biosciences).

### Western Blotting

Mature DCs (mDCs; 1 × 10^6^) were harvested and resuspended in 50 μl lysis buffer (10% glycerol, 2 mM EDTA, pH 8, 137 mM NaCl, 10 mM sodium phosphate, pH 7.2, 1% NP-40, 2 mM phenylmethanesulfonyl fluoride, 20 mM sodium fluoride). After 20 min on ice, lysates were cleared by centrifugation at 4°C and full speed for 20 min. Protein samples were separated using SDS-polyacrylamide gel electrophoresis (SDS-PAGE) and subsequently transferred onto a nitrocellulose membrane. After blocking with 5% (w/v) dry milk or 1x RotiBlock (Carl Roth, Karlsruhe, Germany) the membrane was incubated with the primary antibody at 4°C overnight. After incubation with the appropriate secondary HRP-labeled antibody, detection was performed using Amersham ECL Prime Western Blotting Detection Reagent (GE Healthcare, Solingen, Germany). The following primary antibodies were used for Western blot analyses: anti-CD83 (clone F-5, Santa Cruz Biotech Inc., Heidelberg, Germany, 1:1000), anti-β-actin (clone AC-74, Sigma Aldrich; 1:2000), anti-GAPDH (clone 6C5, Millipore, Schwalbach, Germany), and anti-CMV pp86 (IE2, clone 12E2, Santa Cruz Biotech Inc; 1:1000).

### ELISA for Detection of Soluble CD83 (sCD83)

Soluble CD83 levels were determined using a sandwich ELISA. Ninety-six-well plates (Maxisorp; Nunc, Thermo Scientific) were pre-coated for 1 h at 37°C with anti-CD83 (clone HB15a, Beckman Coulter, Brea, CA, USA), blocked with 10% goat serum (Life Technologies), and finally samples were added. After an overnight incubation at 4°C wells were incubated with 10 μg/ml rabbit-anti-CD83 (Sigma Aldrich) in diluent (5% goat serum, 5% mouse serum, and 1% non-fat dry milk in PBS) for 1 h at 37°C. Next, wells were incubated with goat-anti-rabbit-biotin (Dako, Eching, Germany, 1:5000) for 1 h at 37°C following a 1 h incubation at 37°C with streptavidin-HRP (Dako, 1:2000) in 0.5% BSA in PBS. Between each antibody incubation step, plates were thoroughly washed with 0.1% Tween 20 in PBS. Standard curves for the estimation of sCD83 concentration were generated using serial dilutions of recombinant sCD83 and the results were expressed as a protein concentration based on the protein standard. Plates were developed using BD OptEIA (BD Biosciences) and measured using a Tecan infinite^®^ F500 plate reader.

### CHX-ActD Chase Experiment

Mature DCs were mock-infected or infected with HCMV TB40E/IE2-EYFP at an MOI of 2 in presence of 100 μg/ml CHX. Cells were cultured in RPMI 1640 supplemented with 1% autologous serum, 10 mM HEPES, 2 mM L-glutamine, 100 U/ml penicillin, 100 mg/ml streptomycin, 40 U/ml GM-CSF, 250 U/ml IL-4, and 100 μg/ml CHX. After a 6 h incubation at 37°C, mDCs were washed twice in RPMI 1640 supplemented with either 5 μg/ml ActD or the equal volume of DMSO and cultured for additional 16 h as described above in the presence of ActD or DMSO, respectively. Finally, cells were harvested and surface expression of CD83 was analyzed by flow cytometry. The efficiency of blocking early gene expression was controlled by infection of mDCs with HCMV TB40E/UL84Pr-luc treated in parallel as described above, using a luciferase reporter assay.

### Luciferase Reporter Assay

Luciferase activity of HCMV TB40E/UL84Pr-luc infected mDCs was performed in triplicates using the Dual-Luciferase Reporter Assay System (Promega, Mannheim, Germany) according to the manufacturer’s recommendations. The relative luminescence units (RLU) were determined using the Tecan infinite^®^ F500 plate reader.

### Electroporation of mDC with DNA

Mature DC (3 × 10^6^) were electroporated with 3 μg of the IE2-expression vector pHM134 (Lang et al., 1995) using P3 Primary Cell 4D-Nucleofector^®^ X Kit (Pulse: EH-100) and the 4D-Nucleofector Device (Lonza) according to the manufacturer’s instructions. After electroporation, the nucleovette was immediately flushed with 500 μl warm RPMI 1640 (Lonza) and cells were transferred to a 24-well tissue culture plate. RPMI 1640 supplemented with 1% autologous serum, 10 mM HEPES, 2 mM L-glutamine, 100 U/ml penicillin, 100 mg/ml streptomycin, 40 U/ml GM-CSF, and 250 U/ml IL-4 were added at a final concentration of 0.5 × 10^6^ cells/ml and mDCs were incubated for 18–20 h.

### Statistical Analyses

Results are displayed as means ± standard deviation (SD). For multiple comparisons, data were analyzed using One-Way ANOVA and Bonferroni’s Multiple Comparison *post hoc* test. For comparison of two data sets *p*-values were determined using the student’s *t*-test. Significance was accepted if *p* was <0.05.

### Approvals and Legal Requirements

For the generation of monocyte-derived DCs from leukapheresis products of healthy donors, a positive vote from the local ethics committee has been obtained (reference number 4556). This study was carried out in accordance with the recommendations of the ethics committee of the “Friedrich-Alexander-Universität Erlangen-Nürnberg” with written informed consent from all subjects. All subjects gave written informed consent in accordance with the Declaration of Helsinki.

## Results

Senechal et al. (2004) reported that infection of mDCs with HCMV leads to a loss of CD83 cell surface expression which finally results in a blocked T cell activation. As a mechanism they suggested that a soluble form of CD83 is shed from the cell surface and that this soluble form, which has immunosuppressive properties (Lechmann et al., 2001), is released into the supernatant, however, the precise mode of action was not known (Senechal et al., 2004). Therefore, the aim of this study was to further investigate the mechanisms underlying the down-modulation of CD83 after HCMV infection of mDCs.

**FIGURE 1:**
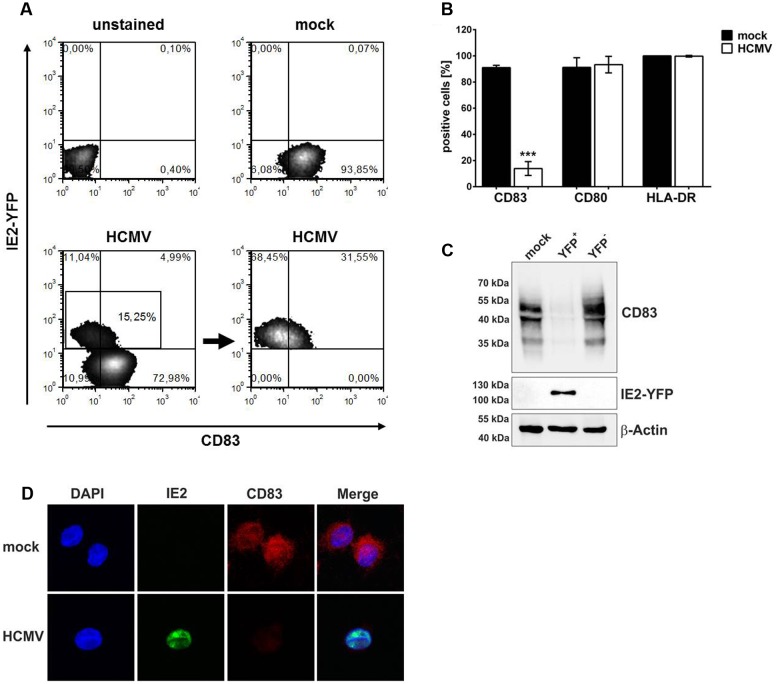
**Human cytomegalovirus infection induces specific CD83 reduction.** Mature DCs (mDCs) were mock- or HCMV-infected. After 16 h, cells were harvested and HCMV-infected as well as mock-infected mDCs were analyzed for their CD83 expression by flow cytometry. **(A)** Gating strategy for flow cytometric analyses. HCMV positive cells were directly analyzed by gating for their IE2-YFP expression. **(B)** Summary of three independent experiments performed with cells of different healthy donors. Cells were stained for their surface expression of CD83, CD80 and HLA-DR (MHC class II). Significant changes are indicated by asterisks (^∗∗∗^*p* < 0.001). **(C)** mDCs were mock- or HCMV-infected. After 16 h of infection cells were harvested and HCMV-positive cells (YFP^+^) were separated from HCMV negative mDCs (YFP^-^) by sorting, based on YFP expression. Subsequently, cells were lysed and analyzed by Western blot for their CD83 expression using a CD83-specific antibody. An IE2-specific antibody was used to verify the purity of the sorted samples and a β-actin antibody was used to assure equal loading. The experiment was performed at least three times and representative data are shown. **(D)** mDCs were mock- or HCMV-infected and after 16 h cells were analyzed for their CD83 expression levels via immunofluorescence. HCMV positive cells were identified based on their IE2-YFP expression. Dapi was used for visualization of the nucleus. One representative experiment out of four is shown.

For the following experiments the recombinant HCMV TB40E/IE2-YFP expressing an IE2-EYFP fusion protein was used (Wagenknecht et al., 2015). This virus strain allows direct analyses of HCMV positive mDCs despite a relatively low infection efficiency of about 15% (**Figure 1A**). In order to analyze whether the loss of CD83 from the cell surface of mDCs after HCMV infection is a CD83 specific effect or due to a general down-regulation of mDC surface proteins, mDCs were either HCMV-infected or were left uninfected. After 16 h of incubation cells were harvested and HCMV positive mDCs were screened for CD83, CD80, and HLA-DR surface expression (**Figure 1B**, white bars) in comparison to mock infected mDCs (**Figure 1B**, black bars) using flow cytometry. While CD83 surface expression was significantly reduced (*p* < 0.001), CD80 and HLA-DR were not influenced by HCMV infection. These data confirmed that CD83 is specifically down-modulated upon infection of mDCs with HCMV.

Senechal et al. (2004) reported that CD83 is lost from the cell surface but intracellular CD83 levels are not influenced. To verify these observations mDCs were mock- or HCMV-infected, and after 16 h of incubation, cells were sorted for HCMV-positive (YFP^+^) and HCMV-negative (YFP^-^) cells. Samples were subjected to Western blot analyses for detection of CD83 expression (**Figure 1C**, top panel). IE2-expression was also analyzed to verify the purity of the sorting process (**Figure 1C**, middle panel) and β-actin was used as loading control (**Figure 1C**, bottom panel). Compared to mock as well as HCMV-negative mDCs (YFP^-^) which show a clear signal for CD83, HCMV-positive cells (YFP^+^) lost CD83 nearly completely. Additionally, immunofluorescence analyses of mock- or HCMV-infected mDCs 16 hpi also showed an overall reduction of CD83 expression levels upon infection (**Figure 1D**). These data indicate that CD83 is not only shed from the cell surface but also lost inside the cell, suggesting an additional mechanism for CD83 down-modulation.

### CD83 Is Degraded via a Proteasome-Dependent Mechanism

**FIGURE 2:**
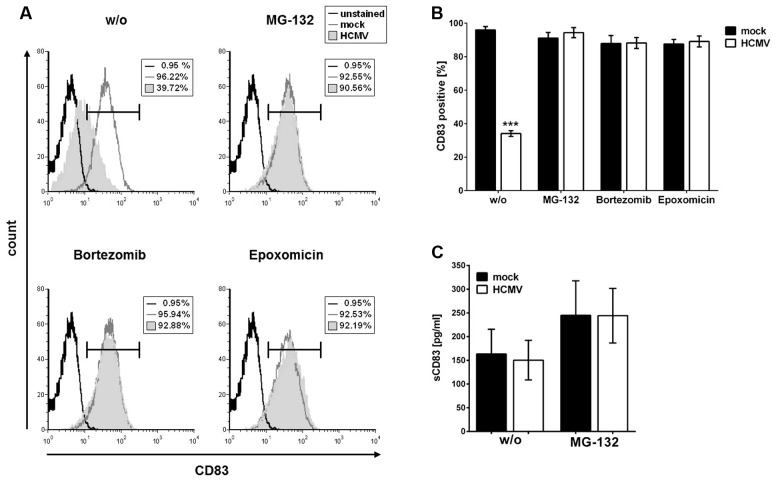
**CD83 down-modulation can be prevented by inhibition of the proteasome.** mDCs were mock- or HCMV-infected. One hour post-infection (hpi) cells were treated either with DMSO or with 10 μM MG-132, 2 μM Bortezomib, or 5 μM Epoxomicin. After additional 16 h of incubation cells were harvested and analyzed for their CD83 surface expression using flow cytometry. **(A)** Results of flow cytometric analyses. Overlays of mock sample (dark gray histogram), HCMV-infected sample (filled gray histogram) and unstained condition (black histogram), as negative control, are shown. The experiment was performed three times and representative data are shown. **(B)** Summary of three independent experiments performed as described above. Mock-infected cells (black bars) were used as controls and treated in the same way as HCMV-infected samples (white bars). Significant changes (^∗∗∗^*p* < 0.001) are indicated by asterisks. **(C)** Detection of soluble CD83 by ELISA in the supernatant of mock- or HCMV-infected mDCs. mDCs were infected as described above and treated with or without 10 μM MG-132 1 hpi. After additional 16 h the supernatant was harvested and analyzed for their soluble CD83 content using ELISA. Mock- and HCMV-infected samples are depicted as black and white bars, respectively.

It has previously been reported that HSV-1-induced CD83 degradation after infection of mDCs can be prevented by inhibition of the proteasome (Kummer et al., 2007). To investigate whether a similar mechanism is responsible for HCMV-induced CD83 down-modulation, infection experiments using proteasome inhibitors were performed. First, mDCs were mock-infected or HCMV-infected and 1 hpi, cells were treated with different proteasome inhibitors (10 μM MG-132, 2 μM Bortezomib, or 5 μM Epoxomicin) or DMSO as control. After 16 h, mDCs were analyzed for their CD83 surface expression by flow cytometry (**Figures 2A,B**). **Figure 2A** shows the results of one representative experiment while **Figure 2B** illustrates the results of three independent experiments performed with cells of different healthy donors. Mock-samples (**Figure 2A**, gray histograms and Figure **2B**, black bars) show no CD83 reduction whereas in the HCMV positive sample without any inhibitor a significant CD83 reduction (*p* < 0.001) was observed. In contrast, treatment with proteasome inhibitors MG-132, Bortezomib or Epoxomicin prevented CD83 down-modulation, demonstrating that CD83 is degraded via the proteasome (**Figure 2A**, gray filled histograms and **Figure 2B**, white bars).

In a next step, we were wondering if the observed proteasomal degradation of CD83 takes place in parallel to the proposed shedding of a soluble form of CD83 (sCD83) from the cell surface. Therefore, we determined the levels of sCD83 in the supernatant of the HCMV-infected mDC cell culture. In sharp contrast to the data reported by Senechal et al. (2004), we observed no difference regarding the sCD83 levels in HCMV-infected samples compared to mock samples. Treatment with MG-132 led to an overall and therefore unspecific increase of sCD83 levels in the supernatant of mock- as well as HCMV-infected cells (**Figure 2C**).

These data indicate that CD83 is not shed from the cell surface of HCMV-infected mDCs but rather degraded via the cellular proteasome.

### An Immediate Early Gene Product Is Responsible for HCMV-Mediated CD83 Degradation

**FIGURE 3:**
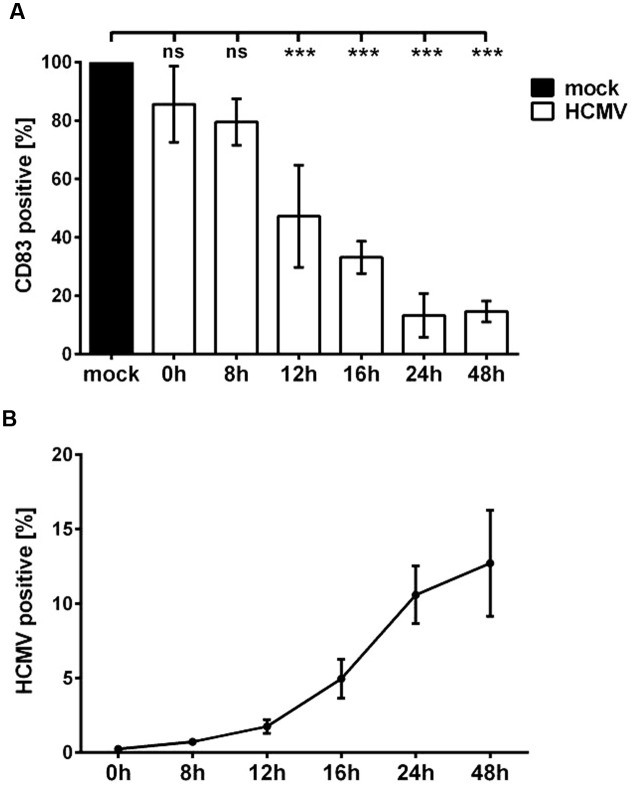
**Significant CD83 down-modulation occurs already 12 h after HCMV infection.** mDCs were mock- or HCMV-infected, harvested at indicated time points and analyzed by flow cytometry. **(A)** DCs were stained for CD83 surface expression. Mock-controls were set to 100% (black bar) and relative CD83 surface expression of HCMV-infected mDCs (white bars) is shown. Significant changes (^∗∗∗^*p* < 0.001) are indicated by asterisks and non-significant changes (*p* > 0.05) are labeled as “ns.” **(B)** At indicated time points cells were analyzed for YFP expression and percentages of HCMV positive mDCs are shown. Experiments were performed three times with cells from different healthy donors.

To further elucidate the mechanism leading to CD83 reduction after HCMV infection of mDCs, we aimed to identify the effector protein/s inducing CD83 down-modulation. To narrow down the phase of the viral replication cycle in which CD83 loss occurs, an infection time course experiment was performed. Therefore, mDCs were mock- or HCMV-infected and analyzed for CD83 expression at different time points post-infection using flow cytometry (**Figure 3A**). HCMV infection efficacy was determined in parallel by measuring YFP-expression (**Figure 3B**). **Figure 3A** illustrates CD83 reduction in HCMV-infected mDCs (white bars) in comparison to the mock control (black bar). After 12 h a significant loss of CD83 (*p* < 0.001) could be observed. At this time point only 50% of the infected cell population showed CD83 expression. CD83 reduction increased over time and after 24 h only 15% of mDCs expressed CD83. This CD83 down-modulation was paralleled by the increase of YFP expression, reflecting expression of IE2 (**Figure 3B**).

Taken together these observations clearly suggest a correlation between IE gene expression and CD83 down-modulation, leading to the hypothesis that the viral effector might be expressed in the IE or early phase of the HCMV replication cycle.

Knowing that a gene product of the IE or early phase is most likely responsible for the down-modulation of CD83, we further wanted to differentiate a possible IE effect from an early effect. The replication cycle of HCMV, as for all herpes viruses, is a strictly regulated cascade of three phases. First, IE gene products are expressed and these IE proteins are required to initiate expression of early (E) genes. The proteins of the E phase are then needed to initiate the expression of late genes. This feature can be used to discriminate between IE and E gene effects performing a cycloheximide-ActD-chase experiment. In brief, mDCs were mock- or HCMV-infected in the presence of DMSO as control or 100 μg/ml cycloheximide (CHX). This CHX treatment prevents protein translation of viral genes but allows gene transcription. After 6 h of incubation, CHX was washed out and cells were incubated in DC-medium, containing either 5 μg/ml ActD or DMSO (as a control) for additional 16 h. ActD allows translation of already existing mRNAs, but prevents the synthesis of new mRNAs. At the end of incubation, cells were harvested and stained for their CD83 surface expression.

**FIGURE 4:**
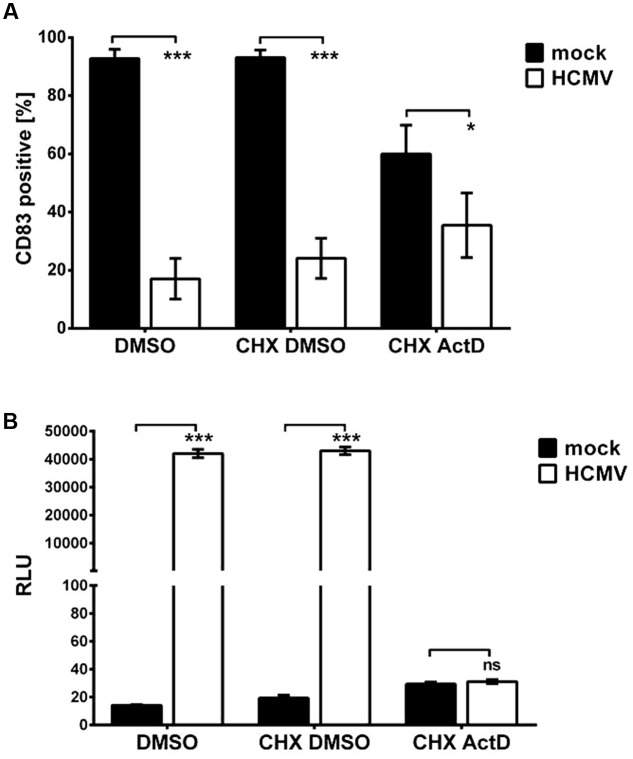
**An immediate early gene product is responsible for the induction of CD83 degradation.** **(A)** mDCs were mock- or HCMV-infected in presence of Cycloheximide (CHX) to inhibit translation or DMSO serving as negative control. Six hpi, CHX was replaced by ActD, inhibiting transcription, or DMSO as control. After an additional 16-h incubation, cells were harvested and analyzed for their CD83 surface expression by flow cytometry. Significant changes are indicated by asterisks (^∗^*p* < 0.05; ^∗∗∗^*p* < 0.001), non-significant changes (*p* > 0.05) as “ns.” Mock controls are depicted as black bars and HCMV-infected samples are shown in white. **(B)** mDCs were infected with HCMV TB40E/UL84Pr-luc to verify the inhibition of early genes during the CHX-ActD-chase experiment. Cells were treated as described above. After 16 h of incubation, cells were harvested and a luciferase assay was performed. The luminescence intensities of mock-samples (black bars) and HCMV-infected samples (white bars) are shown. The experiment was performed three times with cells from different donors and significant changes (^∗∗∗^*p* < 0.001) are indicated by asterisks. Non-significant changes (*p* > 0.05) are depicted as “ns.”

In **Figure 4A** the results of the CHX-ActD-chase experiment are illustrated. Mock controls are depicted in black, HCMV-infected samples in white. CD83 was reduced significantly in CHX-DMSO-treated HCMV positive cells as well as in DMSO-treated cells. In these settings, gene products of all phases are present. In CHX-ActD-treated samples an overall reduction of CD83 surface expression is already observed in mock-infected cells. Nevertheless, there is a significant reduction of CD83 (*p* < 0.05) in HCMV positive CHX-ActD-treated mDCs compared to the mock control indicating that IE gene products are involved in the loss of CD83.

To verify inhibition of the expression of early and late genes by the afore described experiment, cells were infected in parallel with virus strain HCMV TB40E/UL84Pr-luc encoding a luciferase gene under the control of the promoter of the early gene UL84, and treated as described above. At the end of the incubation period a luciferase assay was performed (**Figure 4B**). Mock-infected mDCs served as negative control (**Figure 4B**, black bars). DMSO as well as CHX-DMSO-treated samples show high luciferase activity whereas CHX-ActD-treated mDCs show a luminescence intensity similar to mock-infected cells, confirming that expression of E genes and in turn L genes is inhibited.

### IE2 Is Sufficient to Induce CD83 Down-Modulation

**FIGURE 5:**
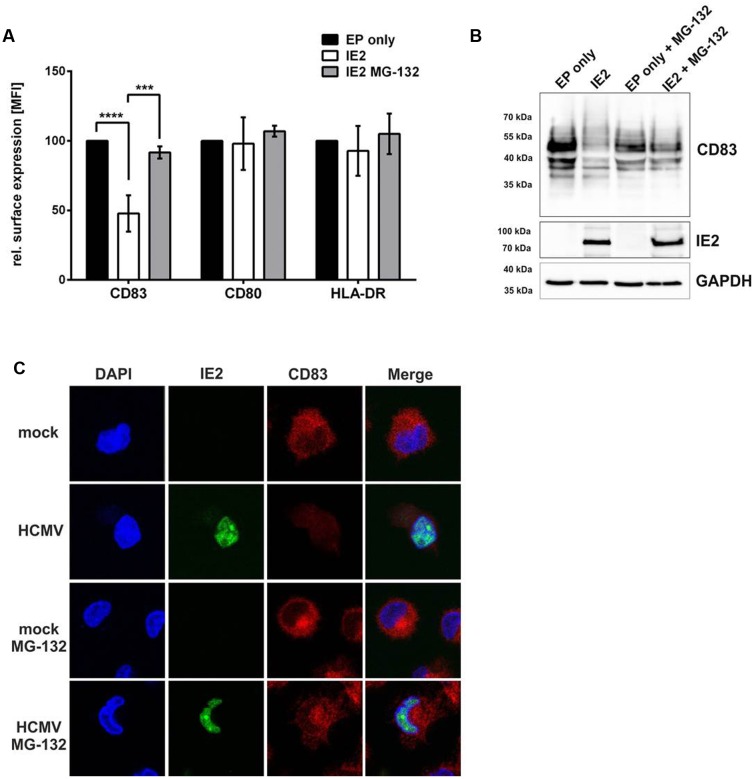
**IE2 is sufficient to induce CD83 degradation in mDCs.** mDCs (3 × 10^6^) were electroporated with 3 μg DNA of an IE2 expressing plasmid (white bars) or were pulsed without addition of DNA as negative control (EP only; black bars). Directly after electroporation mDCs were treated with or without MG-132 and incubated for 16 h. **(A)** Cells were analyzed for their surface expression of CD83, CD80, and HLA-DR using flow cytometry. The experiment was performed at least five times with cells from different healthy donors. Significant changes are indicated by asterisks (^∗∗∗^*p* < 0.001; ^∗∗∗∗^*p* < 0.00001). **(B)** Western Blot analysis for CD83 and IE2 expression. GAPDH was used as internal loading control. The experiment was conducted three times with cells from different donors and representative data are shown. **(C)** mDCs were mock- or HCMV-infected, treated with or without MG-132 and analyzed for their CD83 expression levels using immunofluorescence 16 hpi. The IE2-YFP fusion protein allows direct visualization of IE2 expression as well as localization in HCMV positive cells. The nucleus was visualized using Dapi. The experiment was performed four times and representative data are shown.

Since our data suggested that an IE gene product is responsible for the induction of CD83-degradation, and the knowledge that levels of the IE protein IE2 increased in parallel to the loss of CD83, we next checked whether IE2 is able to induce CD83-degradation. For this purpose mDCs were electroporated with an IE2 expression plasmid and treated with or without MG-132. As mock control, cells were pulsed but without addition of any DNA (EP only). After 20 h, cells were harvested and analyzed via flow cytometry and Western blotting (**Figure 5**). **Figure 5A** illustrates the analysis of CD83 surface expression. IE2 positive cells showed a highly significant (*p* < 0.0001) down-regulation of CD83 surface expression (**Figure 5A**, white bar) which could be prevented by the addition of the proteasome inhibitor MG-132 (**Figure 5A**, gray bar). CD80 and HLA-DR were used as control and were not affected in the presence of IE2. Western blot analysis confirmed the results of flow cytometry analysis and verified expression of IE2 (**Figure 5B**, top and middle panel, respectively). GAPDH was used as loading control (**Figure 5B**, bottom panel).

In order to investigate whether IE2 co-localizes with CD83 in HCMV-infected mDCs, which would suggest that IE2 induces CD83-degradation via a direct interaction, we performed immunofluorescence analyses (**Figure 5C**). Therefore, mDCs were mock- or HCMV-infected, treated with or without MG-132 and analyzed for their CD83 as well as IE2 expression. The treatment of HCMV-infected mDCs with MG-132 restored CD83 expression reaching levels of the respective mock-condition. However, no co-localization of IE2 and CD83 was observed in the context of an HCMV-infection hinting toward an indirect mechanism for IE2-induced CD83-degradation.

Taken together these data clearly demonstrate that HCMV infection of mDCs does not lead to a shedding of a soluble form of CD83 from the cell surface but to proteasomal degradation of CD83. Furthermore, we were able to show that the major IE protein IE2 is sufficient to induce this degradation even in the absence of any additional viral factor.

## Discussion

In the present study, we report that upon HCMV infection of mDCs CD83 surface expression is reduced with fast kinetics, becoming significant already 12 h after infection and reaching its maximum 16 hpi. Interestingly, other mDC surface markers such as CD80 and MHC class II were not affected during the observation period. This is in line with results obtained from HSV-1-infected mDCs in which CD83 is also rapidly down-modulated while the expression of co-stimulatory molecules such as CD80 and CD86 are not influenced (Kruse et al., 2000; Kummer et al., 2007). In addition, it has been described that infection of mDCs with HCMV results in down-regulation of MHC class I as well as MHC class II molecules. This reduction, however, is not detectable until 72 hpi (Benz et al., 2001; Sinzger et al., 2006). CD83 degradation specifically occurs very early after infection supporting the hypothesis that CD83 is involved in mechanisms important for the initiation of antiviral immune responses especially for T cell activation (Kruse et al., 2000; Scholler et al., 2002; Hirano et al., 2006). The fact that several viruses, especially herpesviruses, directly target CD83 points to the importance of this molecule in the establishment of antiviral immunity (Kruse et al., 2000; Morrow et al., 2003; Senechal et al., 2004).

Here, we clearly demonstrated that CD83 is not lost from the cell surface of mDCs upon HCMV infection but that it is rapidly degraded via a proteasome-dependent mechanism. This observation is in sharp contrast to the result of Senechal et al. (2004). They suggested that infection of mDCs leads to a reduction of merely CD83 surface expression most probably caused by shedding of a soluble form of CD83 which in turn might be responsible for the diminished T cell stimulatory capacity (Senechal et al., 2004). However, using several proteasome inhibitors we observed that CD83 levels can be restored nearly completely, demonstrating that CD83 is degraded in a proteasome-dependent manner, as previously described for HSV-1-infected mDCs (Kummer et al., 2007). We additionally analyzed the supernatants of HCMV infected mDCs for sCD83 levels to address the question whether shedding occurs in parallel with degradation of CD83 as a combined mechanism to rapidly down-modulate CD83 from the cell surface. However, we were not able to detect any differences in sCD83 levels released from HCMV infected mDCs compared to mock-treated cells. As several reports demonstrated that inhibition of the proteasome using MG-132 can activate as well as further induce shedding of proteins, e.g., shown for TNF-α receptors (Levine et al., 2005; Peiretti et al., 2005; Ortiz-Lazareno et al., 2008), we analyzed supernatants from HCMV-infected mDCs treated with MG-132 as well. We observed an increased release of CD83 in the supernatant, but again no differences between mock and HCMV-infected mDCs could be detected confirming our conclusion that CD83 is degraded and not shed from the cell surface. The observed discrepancy concerning sCD83 release between the present study and the work performed by Senechal et al. (2004) might be due to the use of different HCMV strains or the analysis at different time points post-infection.

In order to identify a possible viral effector inducing CD83 down-modulation, we first determined the replication phase in which this candidate protein is expressed. A time course experiment performed with HCMV-infected mDCs indicated a viral gene product that is present already at a very early time point after infection. We confirmed this hypothesis using a CHX-ActD-chase experiment in which only IE gene products were expressed. Results obtained from this experiment supported the hypothesis that CD83 reduction occurs during the IE replication phase and that the viral effector inducing CD83 down-modulation is expressed in this phase. Supported by the fact that levels of the IE protein IE2 increased in parallel to the loss of CD83 we analyzed the effect of this protein on CD83 in more detail. By directly expressing IE2 in mDCs we were able to provide evidence, that this IE protein of HCMV is sufficient to induce CD83 degradation in a proteasome dependent manner in absence of any other viral factor.

Regarding the ability of IE2 to induce a degradation mechanism very little is known so far. One study suggests that IE2 directly binds its substrate Mdm2 (Mouse double minute 2 homolog) and thereby facilitates its degradation in a proteasome-independent manner (Zhang et al., 2006). This mechanism can be precluded for CD83 as this protein is degraded via the proteasome. Beyond that, Khan et al. (2014) demonstrated that IE2 is clearly involved in the induction of the proteasomal degradation of connexin43. In addition, our localization studies of CD83 and IE2 (**Figure 5C**) support the hypothesis that IE2 may induce CD83 degradation in an indirect way by upregulating one or more cellular proteins which subsequently target CD83 to the proteasome.

## Conclusion

The results of the present study demonstrate for the first time that IE2 induces the down-modulation of CD83 very early after HCMV infection of mDCs. In contrast to previous studies, we observed an intracellular loss of CD83 which can be prevented by the use of proteasome inhibitors indicating that reduction of CD83 protein levels is mediated by proteasomal degradation.

## Author Contributions

CH, MK, TS, and AS designed the study and the experiments. CH, LG, PM-Z, LK, and ML performed experiments and analyzed and interpreted the data. CH wrote the paper. MK, TS, AS, LG, PM-Z, and ML critically revised the manuscript. All authors approved the final version of the manuscript.

## Conflict of Interest Statement

The authors declare that the research was conducted in the absence of any commercial or financial relationships that could be construed as a potential conflict of interest.
